# Cisplatin Tumor Biodistribution and Efficacy after Intratumoral Injection of a Biodegradable Extended Release Implant

**DOI:** 10.1155/2011/175054

**Published:** 2011-02-27

**Authors:** Ariella Shikanov, Sergey Shikanov, Boris Vaisman, Jacob Golenser, Abraham J. Domb

**Affiliations:** ^1^Department of Medicinal Chemistry and Natural Products, School of Pharmacy, The Hebrew University of Jerusalem, 91120 Jerusalem, Israel; ^2^Department of Urology, Hadassah Medical Center, 91120 Jerusalem, Israel; ^3^Department of Microbiology and Molecular Genetics, The Kuvin Centre for the Study of Infectious and Tropical Diseases, Hadassah Medical School—Faculty of Medicine, The Hebrew University of Jerusalem, 91120 Jerusalem, Israel

## Abstract

Local delivery of chemotherapeutic drugs has long been recognized as a potential method for reaching high drug doses at the target site while minimizing systemic exposure. Cisplatin is one of the most effective chemotherapeutic agents for the treatment of various tumors; however, its systemic toxicity remains the primary dose-limiting factor. Here we report that incorporation of cisplatin into a fatty acid-based polymer carrier followed by a local injection into the solid tumor resulted in a successful tumor growth inhibition in heterotopic mouse bladder tumor model in mice. Platinum concentration in the tumor tissue surrounding the injected implant remained above the therapeutic level up to 14 days after the injection, while the plasma levels were several orders of magnitude lower comparing to systemic delivery. The reported delivery system increased the maximum tolerated dose of cisplatin 5 times compared to systemic delivery, thus potentially improving antitumor efficacy of cisplatin in solid tumor model.

## 1. Introduction

Polymer-based gels are potential carriers that may affect a target tumor while reducing the toxic effects of the loaded drug. Specifically, we suggest that it is possible to optimize their delivery and improve the responsiveness of solid tumors to current chemotherapeutic agents [1]. 

Cisplatin is widely used for the treatment of testicular, bladder, head and neck, small-cell and non-small-cell lung cancers, but it also possesses substantial side effects, such as nephro-, neuro-, and myelotoxicity [2–4]. Polymer-based cisplatin-loaded drug delivery systems such as liposomes [5], polymeric micelles [6], hydrogels [7], polymeric gels [8], and implants [9] provide an opportunity to deliver high, localized doses of drugs for a prolonged period directly into a tumor or at the site of tumor resection. Implanting a biodegradable device loaded with antineoplastic agent in the cavity created by the tumor removal provides high local concentration of the drug, killing the surviving malignant cells. This may also prevent the systemic side effects of chemotherapy that is normally associated with intravenous administration. Injectable device may also provide sustained, controlled delivery of the drug to the malignant tumor. In addition, clinicians can perform debulking of large tumor prior to the surgery by exposing the tumor to large concentrations of the drug [10]. 

Biodegradable polyanhydrides and polyesters are useful materials for controlled drug delivery [11]. In earlier reports we have described the synthesis and various applications of ricinoleic acid-based polyanhydrides as drug carriers [12, 13]. These polymers have hydrophobic backbone with hydrolytically labile anhydride and/or ester that may hydrolyze to dicarboxylic acids and hydroxy acid monomers when placed in aqueous medium. The toxicity, biodegradation, and elimination of polyanhydrides and aliphatic polyesters have been recently reviewed [14, 15]. The fatty acid components of these polymers undergo extensive metabolism in the body and are mainly excreted in the form of carbon dioxide. The *in vitro* and *in vivo* toxicity tests indicate that these polymers are well tolerated by the tissues and can be generally considered as biocompatible [14]. Furthermore, Vaisman et al. [16] evaluated the safety and biocompatibility of ricinoleic acid-based polymer in rats in high doses intramuscularly, subcutaneously, and intracranially. No systemic tissue damage, polymer-related lesions, or abnormalities could be detected in animals.

Herein we report the application of a biodegradable poly (sebacic-co-ricinoleic) acid (P(SA : RA)) polymer for local cisplatin delivery to the solid tumor. Cisplatin can be incorporated by direct mixing with P(SA : RA), which is an injectable fatty acid-based polymer that solidifies in contact with aqueous media [17, 18]. As a result the incorporated drug is released in a sustained manner over a period of days. We hypothesized that cisplatin released from the polymer formulation injected directly in the solid tumor will stop the tumor growth and result in a prolonged survival. Furthermore, we investigated cisplatin local and systemic distribution after single intratumoral injection in heterotopic mouse bladder tumor model.

## 2. Materials and Methods

### 2.1. Materials

Poly (sebacic acid-co-ricinoleic acid ester anhydride) 2 : 8 and 3 : 7 were synthesized as previously described [17]. Cisplatin was purchased from AlfaAesar (MA, USA). All solvents were analytical grade from BioLab (Jerusalem, Israel) or Frutarom (Haifa, Israel) and were used without further purification. MBT (mouse bladder tumor) cells were a generous gift from Professor Ofer Gofrit [19] from Hadassah Ein-Karem Hospital (Jerusalem, Israel). Cell culture medium and fetal calf serum (FCS) were obtained from Biological Industries (Beit-Haemek, Israel).

### 2.2. Preparation of Formulations and In Vitro Drug Release

The formulations of P(SA : RA) 2 : 8 and 3 : 7 with 5% w/w of cisplatin were prepared under sterile conditions by direct mixing of the polymer with the drug at room temperature. The composition was mixed until a smooth paste was obtained. All formulations were aseptically loaded in syringes and tested for sterility in TSB medium [20]. The obtained formulations were injectable semisolid pastes at room temperature. *In vitro* drug release studies were conducted by injecting 20 mg of the pasty formulations sample in a 50 mL phosphate buffer solution (0.1 M, pH 7.4) at 37°C with constant shaking (100 RPM). The paste hardened to a soft solid shortly after addition to the buffer. The release medium was replaced periodically with fresh buffer solution, and platinum concentration in the solution was determined by Inductively Coupled Plasma Mass Spectrometry (ICP-MS, Perkin Elmer SCIEX). The instrument is based on Dynamic Reaction Cell technology (ELAN DRC II) with performance-enhancing Axial Field technology. The validity of the analytical procedure was established through a study of specificity, precision, linearity, and accuracy. The linearity of the analytical procedure was evaluated by plotting the detector response (peak area) against analyte concentration. Linear regression analysis was applied to calculate the slope, intercept, and linear correlation coefficient (*R* ^2^). The limit of detection (LOD) was calculated as signal-to-noise ratio of 3 : 1, and the limit of the quantification (LOQ) was determined as signal-to-noise ratio of 10 : 1. The number of points used in each curve was 6. Calibration curves were obtained by programmed injection of different aliquots (10–45 *μ*L) of a standard solution with increments of 5 *μ*L. The concentration of standard solutions was 10 ppb of platinum in double distilled water, while the linear region was observed at concentration between 0.01 and 100 ppb. All experiments were performed in triplicate.

### 2.3. In Vitro Cytotoxicity

The MBT cells were maintained in monolayer cultures in Dulbecco's modified Eagle's medium containing 10% (v/v) fetal calf serum and supplemented with 200 IU/mL penicillin and 200 *μ*L/mL streptomycin (Beit-Haemek, Israel) in 75 cm^2^ flasks, in humidified 5% CO_2_ in air, incubated at 37°C [21]. 2 × 10^3^ MBT cells in 100 *μ*L of culture medium were seeded in 96-well plates and were incubated for 24 h at 37°C. Cisplatin in vitro toxicity was tested by adding serial dilutions of cisplatin in a volume of 10 *μ*L to the cultured MBT cells. Cytotoxicity of the polymer degradation products was tested by adding 10 *μ*L solution from the blank polymer release study (same sample used as a blank control for the in vitro drug release). Cell proliferation was estimated by ^3^H-thymidine incorporation [22]. All data presented as mean ± STD of triplicate. The data was plotted as a percentage of the data from the control cultures, which were treated identically to the experimental cultures.

### 2.4. In Vivo Cisplatin Toxicity

In a separate study performed to determine maximal tolerated dose (MTD) for the cisplatin-polymer, mice were injected with increasing doses of cisplatin incorporated in the polymer. The dose of 25 mg/kg was chosen as the treatment dose since it was the maximal cisplatin dose at which mice did not show weight loss throughout the study. The dose of 50 mg/kg was determined as (MTD) for cisplatin-polymer formulation, because all mice survived but showed weight loss. The MTD for intraperitoneal delivery was 5 mg/kg, while LD50 for cisplatin in C3H mice is 10 mg/kg [6].

The experiments in mice were approved by the Ethical Committee for Animal Experimentation of the Hebrew University (NIH approval no. OPRR A01-5011).

### 2.5. In Vivo Antitumor Activity

#### 2.5.1. Inoculation of MBT Cells

Inbred 8–10-week old female C3H mice, weighing about 20 g (Harlan Laboratories, Israel) were kept under specific pathogen-free (SPF) conditions and given free access to irradiated food and acidified water throughout the experiment. Mice were injected subcutaneously via a 27-gauge needle in the posterolateral flank with 5 × 10^5^ MBT cells suspended in 0.1 mL RPMI medium. Tumors were measured using caliper every other day, and their volumes were calculated by the formula: length × width × height × 0.523.

#### 2.5.2. Treatment Protocols

The treatment was initiated 10 days after tumor cells inoculation, when the tumor was palpable, and the volume range was between 0.12–0.243 cm^3^. The mice were randomly assigned to one of the three treatment groups or the two control groups (*n* = 10 in each group). Mice in the control groups received either intratumoral injection of 50 *μ*L of the blank polymer or no treatment at all. The first treatment group was injected with 50 *μ*L of a formulation containing 1% cisplatin in P(SA : RA) 2 : 8 (equivalent to 25 mg/kg dose) intratumorally. The second treatment group was treated with intratumoral injection of 0.1 mL cisplatin solution in saline at a concentration of 1 mg/mL which equals 5 mg/kg, and the third group received intraperitoneal (IP) injection of 0.1 mL of cisplatin solution in saline at a concentration of 1 mg/mL (LD50 for cisplatin in C3H mice is 10 mg/kg). Mice received a single injection during the experiment. The animals were sacrificed when the tumor volume reached 3–3.5 cm^3^.

### 2.6. Platinum Distribution in Plasma and Tissue

Additional group of mice (*n* = 36) was injected with 5 × 10^5^ MBT cells to induce subcutaneous tumor. However, to determine platinum distribution from the injection site in the tumor mass, the treated tumors should partially escape from the treatment; otherwise, there would be no tumor mass to determine the platinum concentration. Thus, 50 *μ*L of the injectable polymer/cisplatin formulation containing 1% cisplatin was injected 13 days after tumor cells inoculation when the tumors volume was >1.2 cm^3^. In this case the tumor could not be totally eliminated, and the pattern of platinum distribution in the tumor could be studied. At different time points (1, 2, 3, 5, 7, and 14 days after injection), six mice were sacrificed. The tumor was excised and frozen at *‒*20°C, and the blood was collected from cardiac puncture, heparinized, and centrifuged (2500 rpm, 5 min) to obtain plasma. The obtained plasma was separated and kept frozen at *‒*20°C. Tissue samples were embedded (O.C.T. Compound, Tissue-Tek, Redding, CA) and sectioned into 50–100-*μ*m-thick sections in a cryostat at *‒*20°C. The sections were weighed, decomposed in nitric acid, and diluted in DDW to obtain 100-, 1000-, and 2200-fold dilutions to determine platinum concentrations in solution and plasma in ICP-MS (Perkin Elmer). All sections were inspected for the presence of nondegraded formulation, and the formulation was manually scooped out to avoid biased calculation of the actual drug concentration in the tissue.

### 2.7. Macroscopic and Histopathological Evaluation

For histopathological evaluation animals were sacrificed 5 days after treatment application, and tumors were dissected and fixed in 4% formaldehyde solution. The tissue was processed into paraffin, and 3-*μ*m sections were stained with hematoxylin & eosin for histological evaluation. The examination parameters included necrosis total area, inflammatory cell infiltration, and intact tumor tissue.

### 2.8. Statistical Analysis

All results are expressed as mean ± standard deviation (STD) of the mean and statistically analyzed using GraphPad Instat ANOVA. *P* values less than  .01 were considered significant for all tests.

## 3. Results

### 3.1. In Vitro Cisplatin Release

**Scheme 1:**
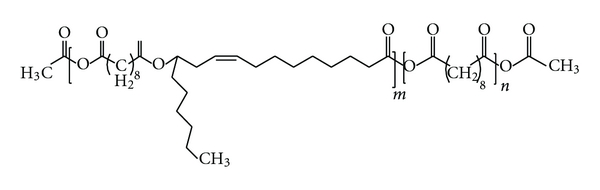
Structure of poly(sebacic acid-co-ricinoleic acid).

**Figure 1:**
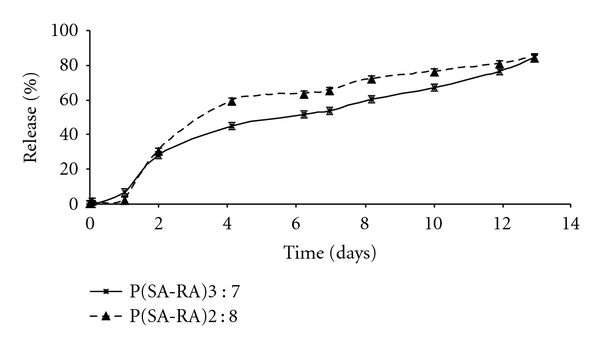
*In vitro* cumulative release of Pt from P(SA : RA)2 : 8 (triangles, dashed line) and P(SA : RA)3 : 7 (stars, solid line) loaded with 5% w/w cisplatin. Each point represents the mean value ± STD (*n* = 3). Release was conducted in 0.1 M phosphate buffer, pH 7.4, at 37°C. Pt concentrations were determined by ICP-MS.

The polymer carriers—P(SA : RA) 2 : 8 and 3 : 7 having molecular weight (Mw) ranging from 4000 to 6000 Da were prepared from RA and SA by melt condensation as previously described [17]. The polymer structure is shown in Scheme 1. Incorporation of 5% w/w of cisplatin in the polymer by mixing at room temperature did not affect the molecular weight of the polymer and had no chemical interaction with the polymer, as was confirmed by GPC and ^1^H-NMR. Cisplatin release from P(SA : RA) had no initial burst effect, and during the first day only 5–7% of the incorporated drug was released (Figure 1), followed by additional 25% on the next day, and reaching 85% in the following ten days.

### 3.2. In Vitro Cytotoxicity

**Figure 2:**
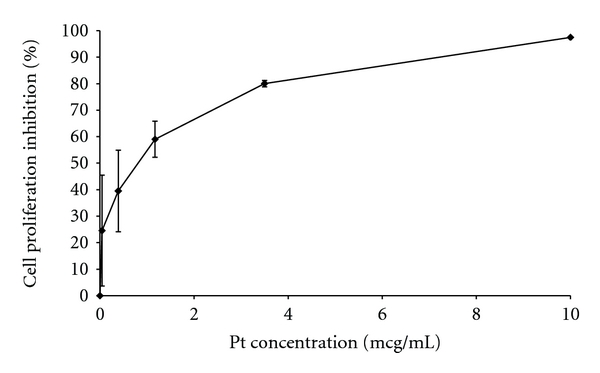
In vitro inhibition effect of cisplatin on MBT cells. Cisplatin at increasing concentrations was added 24 hours after cell incubation in wells.

Growth inhibition of cisplatin in MBT cell culture is shown in Figure 2. The IC_50_ value of cisplatin was found to be 0.8 *μ*g/mL. Similar results of IC_50_ for cisplatin were reported earlier: 4.8 *μ*g/mL for MBT-2 cells [6] and 1.5 *μ*g/mL for Meth-AR-1 cells [7].

### 3.3. In Vivo Antitumor Activity

**Figure 3:**
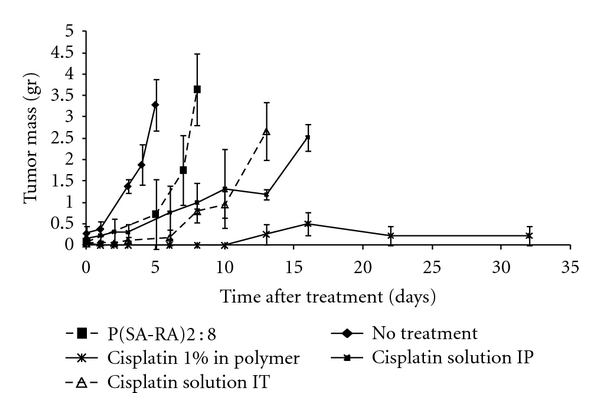
Effect of cisplatin on MBT tumor growth in s.c. implanted mice (*n* = 10). Cisplatin 1% w/w in polymer, 50 *μ*L (∗: solid line); blank polymer (■, dashed line); cisplatin solution (0.1% w/v in saline, 100 *μ*L injection, 5 mg/kg) (∆, dashed line) were injected intratumorally. No treatment group is designated as (♦) with solid line, and the group treated IP with cisplatin solution (1% w/v in saline, 100 *μ*L injection, 5 mg/kg) is designated as (■) with solid line. The tumor volume is expressed as mean ± STD. Statistically significant differences between the groups are signed with a star (*P* < .05,  *).

The efficacy of cisplatin delivered intratumorally was investigated in a heterotopic mouse bladder tumor (MBT) model. The treatment was initiated on the tenth day after tumor cell inoculation. Mice that were not treated and mice injected with the blank polymer were sacrificed 15 and 18 days, respectively, after tumor cells inoculation when the tumor volume exceeded 3.5 cm^3^. However, the growth rate of the tumor was slower in mice injected with the blank polymer that caused statistically significant (*P* < .005, ANOVA) delay in tumor progression (Figure 3). The injection of blank polymer into the tumor damaged its structure and delayed its development, but since there was no therapeutic effect on the tumor cells, as was confirmed in *in vitro *cultured cells, the tumor recovered from the physical injury and continued to grow. The injected dose of cisplatin was the same in intratumoral (IT) and intraperitoneal (IP) injection of cisplatin solution (0.1% w/v in saline, 0.1 mL injection, 5 mg/kg); however, IP delivered drug-inhibited tumor growth more efficiently comparing to IT application of solution. After IT injection of cisplatin solution tumors reached the volume of 3 cm^3^ 13 days after treatment while after IP treatment only after 16 days. Thus, soluble cisplatin delivered in solution either IT or IP showed efficiency compared to nontreatment and blank polymer groups in prolonging mice life. In the cisplatin/polymer group, mice were injected intratumorally with 50 *μ*L of the 1% cisplatin formulation 10 days posttumor inoculation. In 8 mice out of 10 the tumors completely disappeared during the first 10 days after treatment, and mice remained tumorless till the end of the study (40 days posttumor cells inoculation), while, in the other two mice in this treatment group twenty days posttreatment administration, regrowth of a small nodule at the edge of the original tumor appeared, but its dimensions did not increase above 0.3 cm^3^ till the end of the study (40 days posttumor cells inoculation).

### 3.4. Platinum Tumor Distribution

In this study we measured total platinum levels rather than intact cisplatin. Cisplatin is not stable in biological fluids and undergoes ligand-exchange reaction that result in metabolites with different biological activity. Although determining concentrations of both intact cisplatin and its various metabolites would be a more accurate way to predict its activity, measuring total serum platinum is traditionally used for clinical pharmacokinetics assays [8].

**Figure 4:**
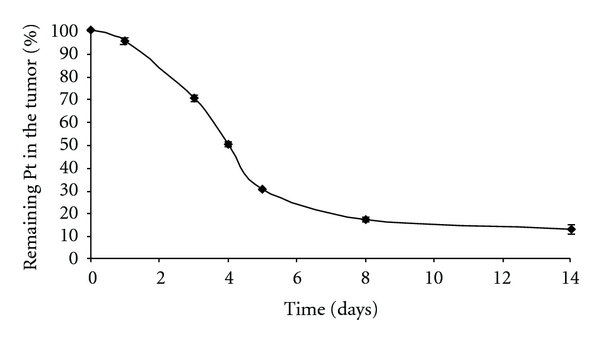
The time profile of platinum remaining in the tumor after intratumoral injection of cisplatin/polymer formulation (1% w/w, 50 *μ*L). Values are expressed as mean ± STD (*n* = 6).

Platinum content in the tumor mass gradually decreased over the time course of 14 days (Figure 4), while 90% of the injected platinum was found in the tumor tissue 24 hours after the injection decreasing to 12% of the injected platinum at 14 days.

**Figure 5:**
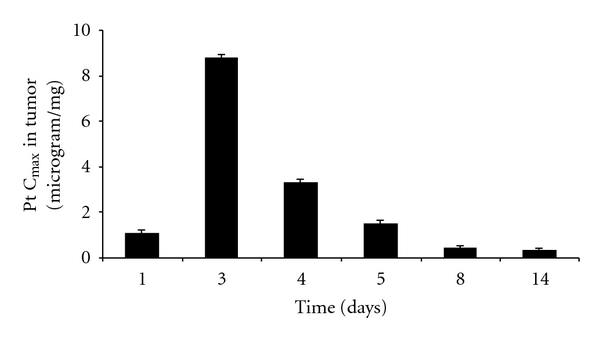
The time profile of platinum maximal concentration (*C*_max_) in the tumor tissue after intratumoral injection of cisplatin/polymer formulation (1% w/w, 50 *μ*L). Values are expressed as mean (*n* = 6).

The peak platinum concentrations (*C*_max_) in tumor tissue at different time points were defined as the amount of platinum per milligram tumor tissue excluding non-degraded formulation (Figure 5). In the first day after injection, platinum concentration in the tumor tissue was still low, which corresponded with the in vitro release results. However, three days after the intratumoral injection, platinum concentration was the greatest and reached 8.8 *μ*g/mg, while platinum concentration in plasma was only 0.12 *μ*g/ml. *C*_max_ gradually decreased in the following days, and 14 days after the injection platinum concentration in tumor was 0.3 *μ*g/mg that is still high enough to induce cytotoxic effect [23]. For comparison, systemic injection of 10 mg/kg free cisplatin produced tumor platinum concentration of 0.014 *μ*g/mg [24], which is lower than *C*_max_ 14 days after intratumoral injection of cisplatin/polymer formulation.

**Figure 6:**
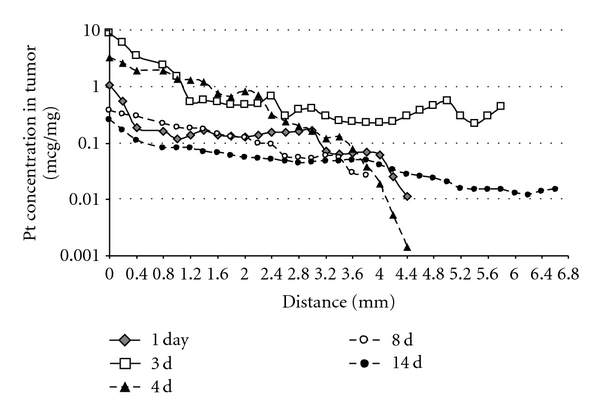
Cisplatin tumor tissue distribution after intratumoral injection of cisplatin/polymer formulation (1% w/w, 50 *μ*L). Each curve represents a different time point when the mice were sacrificed and their tumors processed. Values are expressed as mean (*n* = 6).

The cisplatin/polymer formulation was injected into the center of the tumor; therefore, platinum distribution is expected to be radial, while the greater concentrations are found at the injection site with gradual decrease toward the tumor boarder. Each line in Figure 6 represents platinum concentration pattern at one time point from the center of the tumor toward the edge over the distance in millimeters. After 24 hours subsequent to injection, the maximal platinum concentration of 1.1 *μ*g/mg tumor tissue was found close to the injection site, and it decreased gradually to 0.01 *μ*g/mg at a distance of 4.4 mm from the injection site that is still considered above the therapeutic level. The greatest platinum tumor tissue concentration was found at 3 and 4 days after the injection, reaching 8.8 *μ*g/mg and 3.8 *μ*g/mg, respectively. After 14 days, platinum levels at the injection sites were still above the therapeutic level. Interestingly, in the first 4 days after the treatment, greater gradient was observed between the injection site and the distant areas of the tumor. However, at the later period, the platinum content in the tumor tissue was more equally distributed that can be explained by the drug diffusion and the clearance from the injection site.

### 3.5. Platinum Plasma Levels

**Figure 7:**
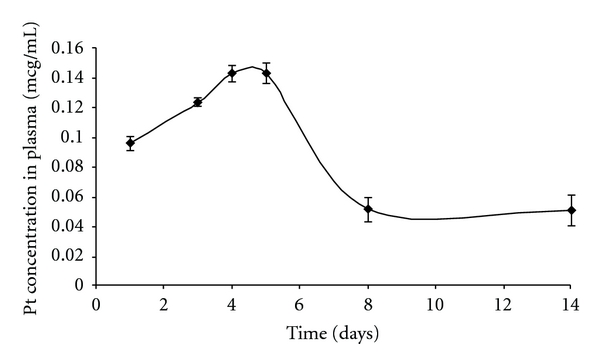
Platinum (Pt) levels in plasma. Pt levels in mice plasma were determined by ICP-MS. Each data point represents the average of six mice ± STD.

As reported elsewhere [6], the plasma platinum levels after IV administration of free cisplatin were 11.7 *μ*g/mL at time zero and decreased during the following 12 hours to 1 *μ*g/ml. After intratumoral injection of cisplatin/polymer formulation platinum plasma levels gradually increased from less than 0.1 *μ*g/mL after 24 hours and peaked at 0.15 *μ*g/mL on days 4–6, followed by sharp decrease to 0.06 *μ*g/mL on day 8 (Figure 7). The platinum plasma levels are closely related to the events occurring in the tumor, where the polymer releases the incorporated drug. Importantly, platinum plasma levels after intratumoral polymer injection were several orders of magnitude lower comparing to systemic delivery at all time points.

### 3.6. Macroscopic and Histopathological Evaluation

**Figure 8:**
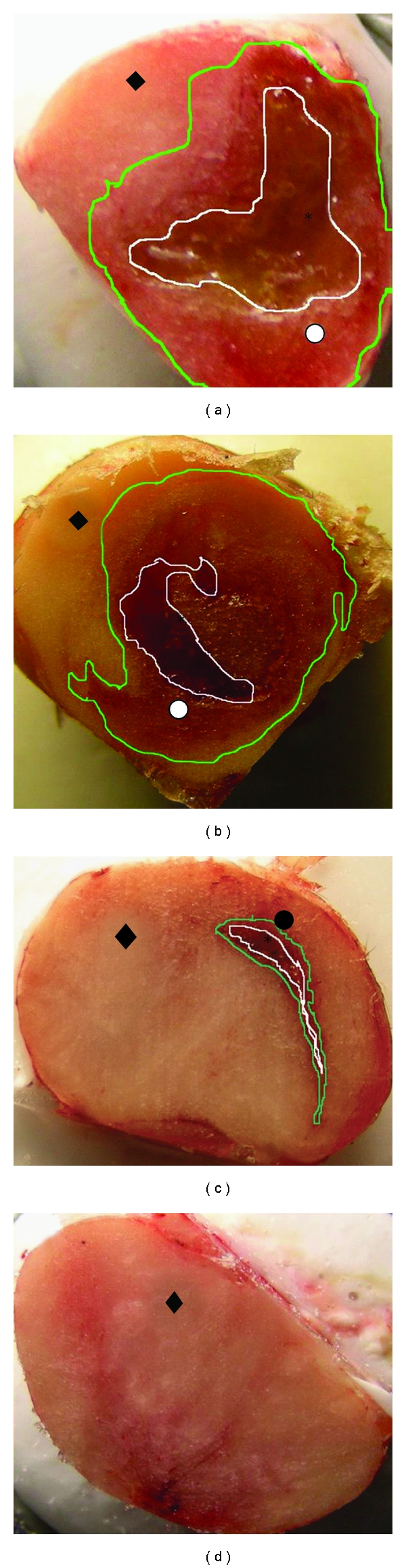
Macroscopic view of frozen tumor tissues at cryostat sectioning: (a) tumor treated with cisplatin/polymer formulation and excised 3 days after injection; (b) tumor treated with cisplatin/polymer formulation and excised 7 days after injection; (c) tumor treated with the blank polymer and excised 3 days after injection; (d) untreated tumor. The polymeric formulation is assigned with the star (∗), the necrotic tissue with the white circle (*⚪*), the infiltration of the inflammation cells with a black circle (•), and the intact tumor cells with a black rhomb (♦).

Figure 8 shows the macroscopic view of the tumors dissected from mice and sectioned in cryostat.  Figure 8(a) shows the MBT tumor treated with 50 *μ*L of 1% w/w cisplatin/polymer formulation and removed 3 days after treatment. The formulation's color is yellow because of the cisplatin color and can be easily recognized (designated as (*), and contoured with white line). Three days after formulation injection, large portion of the injected formulation was still found in the center of the tumor tissue, and the formulation did not totally degrade. A region of a reddish tissue surrounded the injected formulation, which was histologically proven to be a necrotic/inflammatory process (designated as (*⚪*) and contoured with green line), followed by the intact tumor cells (designated as (♦)), as was proved in the histopathological evaluation of the tissue. Seven days after the formulation injection, 20% of platinum was still found in the tumor tissue, but the formulation has already degraded, and the injection site remained free of the polymer and tumor cells (Figure 8(b)). Around the injection site, the necrotic region increased in size, which applies to continuous cytotoxic activity (designated as (*⚪*) and contoured with green line), followed by the intact tumor cells (designated as (♦)). Blank polymer was injected into the tumors and sectioned similarly to eliminate the possibility of the blank polymer cytotoxic activity.  Figure 8(c) shows the tumor with the blank polymer 3 days after injection. The polymer region is surrounded by a mild inflammation region followed by tumor cells, and while partial effect of the polymer on the tumor cells cannot be excluded it was less vigorous than with cisplatin. For comparison, Figure 8(d) shows the tumor without treatment. 

**Figure 9:**
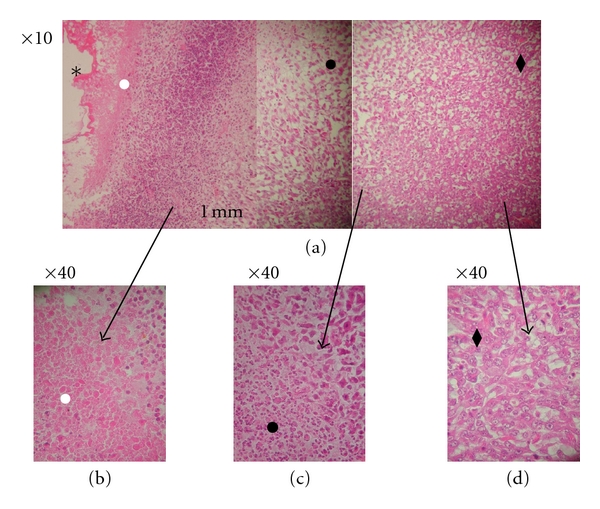
Histology of the MBT tumor injected intratumorally with cisplatin/polymer formulation (1% w/w, 50 *μ*L). (a) Magnification ×10, panoramic view of the slice; (b) magnification ×40, enlargement of the cisplatin/polymer region and the surrounding necrotic area; (c) magnification ×40, enlargement of the border between the end of the necrotic area and start of the intact tumor area; (d) magnification ×40, enlargement of the intact tumor area beyond the effect of cisplatin. The polymeric formulation is assigned with the star (∗), the necrotic tissue with the white circle (*⚪*), the infiltration of the inflammation cells with a black circle (•), and the intact tumor cells with a black rhomb (♦).

**Figure 10:**
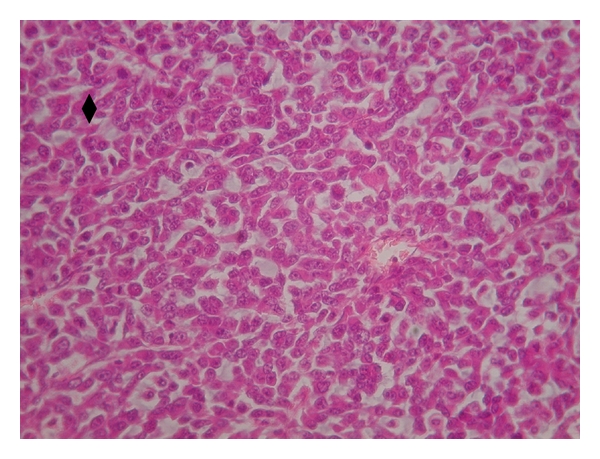
Histology of nontreated MBT tumor (magnification ×40), intact tumor cells.

**Figure 11:**
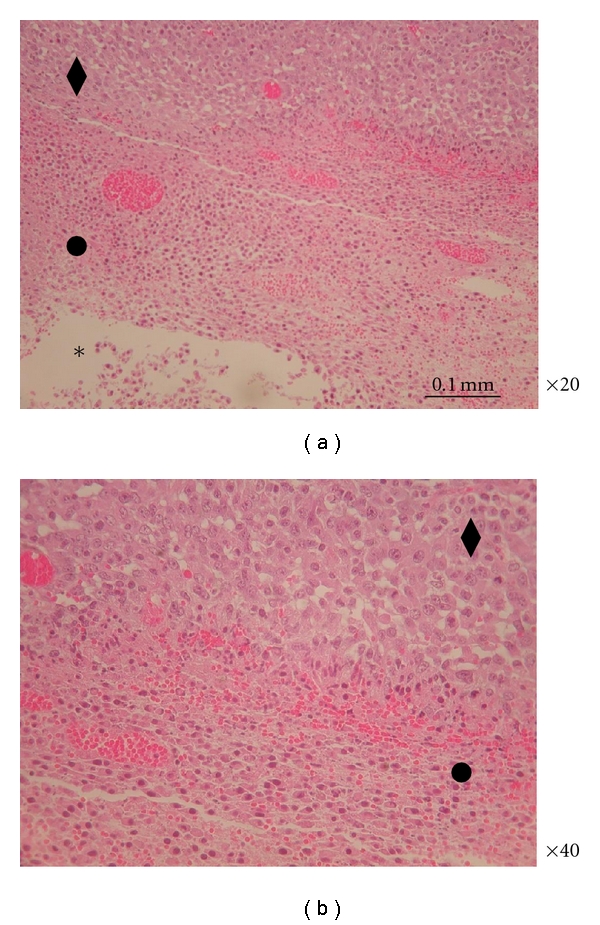
Histology of the MBT tumor injected intratumorally with blank polymer. (a) Magnification ×20; (b) magnification ×40, enlargement of the blank polymer and the surrounding mild inflammation area; bar: 0.1 mm; the polymeric formulation is assigned with the star (∗), the necrotic tissue with the white circle (*⚪*), the infiltration of the inflammation cells with a black circle (•), and the intact tumor cells with a black rhomb (♦).

The changes appearing in the tumor tissue at the formulation injection site and in more distant regions are shown in the panoramic view of the histology images of the tumors injected with cisplatin/polymer formulation (Figure 9(a)). Only necrotic cells are present at the formulation-tumor interface (higher magnification, Figure 9(b)). Along with the necrosis progression, inflammation process is evident, and the region of dead tumor cells extends up to 3.5 mm from the cisplatin formulation. Figure 9(c) is a higher magnification of a border region between the necrotic process and the unaffected intact tumor tissue, followed by mainly tumor cells (Figure 9(d)). The region in the tumor where cisplatin did not diffuse and was below therapeutic dose (Figure 9(d)) has similar cells appearance as in the nontreated tumor (Figure 10). Importantly, tumor cells density became noticeably lower in both necrotic regions and the region beyond the border, which results in enhanced drug penetration and the cytotoxic effect [25].  Figure 11 shows the appearance of the MBT tumor without treatment; no necrosis or inflammation process was evident in the tumor tissue. 

Efficacy studies showed that intratumoral injection of a blank polymer caused a delay in tumor growth (Figure 3). Histological evaluation of the tumors injected with the blank polymer revealed a mild inflammatory reaction and no infiltration region around the polymer for 0.3 mm. Beyond the 0.3 mm, intact tumor cells appear again, as shown in Figures 11(a) and 11(b).

## 4. Discussion

The toxicity of conventional systemic cancer chemotherapy has severely limited the safety and effectiveness of such therapy, and its impact on the quality of life of patients hampers its wider clinical application [8]. Plasma concentrations are often used as a marker of cytotoxic exposure; however, drug delivery to the tumor is determined not only by plasma concentrations but also by distribution from plasma into the extracellular fluid (ECF) of the tumor and from the ECF into the cells. Solid tumors have several potential barriers to drug delivery that may limit drug penetration, such as alteration of distribution of blood vessels, blood flow, interstitial pressure, and microcirculation in the tumor [23]. Therefore, high systemic levels of the cytotoxic drug often cause systemic toxicity without reaching effective concentrations in the tumor. 

Various injectable drug delivery systems have been investigated for local delivery of cisplatin and other anticancer agents. Intradose, a collagen gel, loaded with cisplatin and epinephrine, has been shown to be effective for the treatment of head and neck and hepatocellular cancers [26]. Intradose is an injectable gel that releases cisplatin after intratumoral injection. However, the semiliquid phase of the collagen gel released its content in short time period of hours till couple of days that evokes a need for repeated administrations. Another injectable biodegradable PEG, PLGA polymer system for intratumoral chemotherapy, is Atrigel. This system has a similar drug release period; however, the solidification mechanism involves solvent displacement that can result in higher systemic toxicity. Interestingly, platinum serum levels in rats were 10 times higher after Atrigel injection [8] comparing to the mice serum levels reported in this study, hence presenting lower systemic toxicity of the described formulation combined with local efficiency again tumor cells.

Poly (sebacic co-ricinoleic acid) 2 : 8 used in this study is a hydrophobic polymer, built of natural fatty acids, which can be used for the release of hydrophobic or hydrophilic drugs. The polymeric paste formulation with cisplatin is injectable through a 23-gauge needle and it forms a gel in contact with body fluids by a mechanism that does not involve solvent leaching or temperature change [17, 18]. The purpose of this study was to evaluate the effect of cisplatin-polymer formulation injected intratumorally in heterotopic model in mice compared to immediate release formulation after IP and IT delivery. Interestingly, we noticed that IP cisplatin delivery delayed tumor growth more efficiently compared to the same solution delivered IT. A possible explanation of the superior efficiency in tumor treatment of IP delivery is that the drug was delivered through the blood supply to the tumor and had better chances reaching all the tumor regions, while in the direct injection of cisplatin solution to the tumor the delivery is limited to the injection site, and cisplatin penetration can be inhibited by intratumoral interstitial pressure [27]. Moreover, cisplatin in solution is cleared almost immediately leaving other regions of the tumor untreated. In contrast to IP or IT delivery of cisplatin solution, the described polymeric formulation formed a semisolid implant in situ as was confirmed after tumor excision and sectioning [18]. Because of the hydrophobic nature of the polymer, there was no burst effect upon the formulation injection, and the drug was released at controlled rate, as was determined by plasma platinum levels monitoring. The levels of platinum in the tumors treated with cisplatin/polymer formulation were above the therapeutic threshold during two weeks after the injection, without any signs of systemic toxicity. Importantly, incorporation of cisplatin in the polymer that delivered the drug over prolonged period of time allowed increasing MTD 5 times compared to immediate release formulation, hence injecting 25 mg/kg in polymer versus only 5 mg/kg in solution. Thus, the effectiveness of the polymeric formulation in treating MBT tumor in the described heterotopic model can be the result of higher dose, slow prolonged release and exposure to the drug, and greater surface area of contact between the tumor and the formulation.

Cisplatin is not stable in biological fluids and undergoes ligand-exchange reaction that results in aquated cisplatin that gradually is transformed to other metabolites through reaction with glutathione, albumin, and nucleotides. Although both intact cisplatin and its metabolites are biologically active, it is the intact cisplatin that is responsible for nephrotoxicity. Thus, it can be assumed that systemic platinum levels found after local delivery of cisplatin in the polymer formulation are mainly in the form of biological metabolites, rather than unmodified cisplatin, because of the longer time cisplatin is exposed to biological fluids [24, 28]. 

Moreover, P(SA : RA) has been shown to be effective in treating solid tumors with paclitaxel, which is highly potent hydrophobic chemotherapeutic drug [29, 30]. P (SA : RA)-based polymeric system is unique because it can serve as a vehicle for delivery of both hydrophilic and hydrophobic drugs concomitantly in a single injection, thus increasing the therapeutic potential of the formulation [31–33].

## 5. Conclusion

The results of this work indicate that treatment with the polymer formulation of cisplatin had a positive outcome and inhibited the growth of the tumors. Distribution studies of cisplatin after intratumoral injection showed high and effective concentrations in the tumors. Histological studies proved the existence of the necrotic process caused by the cytotoxic drug.
